# Mesenchymal stromal cells enhance the engraftment of hematopoietic stem cells in an autologous mouse transplantation model

**DOI:** 10.1186/s13287-015-0155-5

**Published:** 2015-09-07

**Authors:** María Fernández-García, Rosa M. Yañez, Rebeca Sánchez-Domínguez, Miriam Hernando-Rodriguez, Miguel Peces-Barba, Guadalupe Herrera, Jose E. O’Connor, José C. Segovia, Juan A. Bueren, María L. Lamana

**Affiliations:** Hematopoietic Innovative Therapies Division. Centro de Investigaciones Energéticas, Medioambientales y Tecnológicas (CIEMAT) and Centro de Investigación Biomédica en Red de Enfermedades Raras (CIBER-ER), Madrid, Spain; Unidad Mixta de Terapias Avanzadas. Instituto de Investigación Sanitaria Fundación Jiménez Díaz. (IIS-FJD, UAM), Avda Complutense 40, 28040 Madrid, Spain; Cytometry Service. UCIM. INCLIVA-Universidad de Valencia, Avda Blasco Ibañez 13, 46010 Valencia, Spain; Laboratory of Cytomics. Universidad de Valencia, Avda Blasco Ibañez 13, 46010 Valencia, Spain

## Abstract

**Introduction:**

Studies have proposed that mesenchymal stem cells (MSCs) improve the hematopoietic engraftment in allogeneic or xenogeneic transplants and this is probably due to the MSCs’ immunosuppressive properties. Our study aimed to discern, for the first time, whether MSC infusion could facilitate the engraftment of hematopoietic stem cells (HSCs) in autologous transplantations models, where no immune rejection of donor HSCs is expected.

**Methods:**

Recipient mice (CD45.2) mice, conditioned with moderate doses of radiation (5-7 Gy), were transplanted with low numbers of HSCs (CD45.1/CD45.2) either as a sole population or co-infused with increasing numbers of adipose-derived-MSCs (Ad-MSCs). The influence of Ad-MSC infusion on the short-term and long-term engraftment of donor HSCs was investigated. Additionally, homing assays and studies related with the administration route and with the Ad-MSC/HSC interaction were conducted.

**Results:**

Our data show that the co-infusion of Ad-MSCs with low numbers of purified HSCs significantly improves the short-term and long-term hematopoietic reconstitution of recipients conditioned with moderate irradiation doses. This effect was Ad-MSC dose-dependent and associated with an increased homing of transplanted HSCs in recipients’ bone marrow. *In vivo* and *in vitro* experiments also indicate that the Ad-MSC effects observed in this autologous transplant model are not due to paracrine effects but rather are related to Ad-MSC and HSC interactions, allowing us to propose that Ad-MSCs may act as HSC carriers, facilitating the migration and homing of the HSCs to recipient bone marrow niches.

**Conclusion:**

Our results demonstrate that Ad-MSCs facilitate the engraftment of purified HSCs in an autologous mouse transplantation model, opening new perspectives in the application of Ad-MSCs in autologous transplants, including HSC gene therapy.

## Introduction

Mesenchymal stem cells (MSCs) are fibroblast-like cells capable of differentiating into different cell lineages [1–3] and of exerting immunosuppressive properties through their interaction with the innate and adaptive immune system [4–7]. MSCs also play an important role in the bone marrow (BM) niche by secreting components of the extracellular matrix, cytokines, and growth factors, all of which are essential for hematopoietic stem cell (HSC) maintenance and differentiation [2, 3].

The unique immunosuppressive properties of MSCs have led to their clinical application for the treatment of several inflammatory diseases, mainly graft-versus-host disease (GVHD) in allogeneic HSC transplants (HSCTs) [8, 9] but also in autoimmune diseases such as multiple sclerosis [10, 11] and Crohn’s disease [12, 13] among others. Additionally, several MSC-based therapies have been applied in the field of regenerative medicine [14–17]. Significantly, MSCs have not generated any severe adverse side effects in any of their clinical applications, proving the safety of their use [18].

In addition to their application in the abovementioned clinical settings, MSCs have been used to facilitate the engraftment of HSCs, both in experimental transplantation models and in clinical applications. In experimental models, MSCs improved the engraftment of human CD34^+^ cells transplanted into non-obese diabetic/severe combined immunodeficiency (NOD/SCID) mice [19, 20]. In humans, MSCs have been used in allogeneic transplants to limit risks of graft failure [21–23].

The HSC engraftment effect mediated by MSCs in xeno- or allogeneic transplants might be attributed to the immunosuppressive properties of the MSCs. However, in autologous HSC transplants, in which no immune reaction between donor and host tissues is expected, only one pilot study was performed in patients with advanced breast cancer, which suggested that MSCs may accelerate HSC engraftment and platelet recovery after high-dose chemotherapy and HSC rescue. Although the feasibility and safety of MSC co-infusion were demonstrated in this study, the absence of a control group limited their conclusions [24].

In spite of the studies conducted so far, it is currently unknown whether MSCs would be able to facilitate the HSC engraftment in an autologous transplantation context. A more direct effect of MSCs favouring the HSC engraftment moved us to explore this possibility. We reasoned that if this was the case, MSCs would have a significant value in HSC gene therapy applications to facilitate the engraftment of gene-corrected HSCs.

To achieve this aim, in the current studies, we used a congenic CD45.1 and CD45.2 mouse transplantation model, in which mouse adipose-derived MSCs (Ad-MSCs) were co-transplanted with purified HSCs into recipient mice. Our data demonstrate, for the first time, the relevance of Ad-MSCs to facilitate the stable engraftment of HSCs in an autologous transplantation model. Significantly, this effect was most evident when limiting engraftment conditions (i.e., low numbers of HSCs and mild conditioning regimes were used), opening new perspectives to the use of MSCs in hematopoietic gene therapy.

## Methods

### Mice

B6D2F1 (H2^b/d^, CD45.2), P3D2F1 (H2^b/d^, CD45.1/CD45.2), mice, aged 10–12 weeks, were housed and bred at the CIEMAT (Centro de Investigaciones Energéticas, Medioambientales y Tecnológicas) Laboratory Animals Facility (registration number ES280790000183) from breeding pairs originally obtained from The Jackson Laboratory (Bar Harbor, ME, USA). Mice were routinely screened for pathogens, in accordance with FELASA (Federation of European Laboratory Animal Science Associations) procedures, and received water (50 m filtered and ultraviolet irradiated) and food (SAFE R04 25 KGy gamma-irradiated) *ad libitum.* All experimental procedures were carried out in accordance with Spanish and European regulations (Spanish RD 53/2013 and Law 6/2013 that transposes and fulfill the European Directive 2010/63/UE about the use and protection of vertebrate mammals used for experimentation and other scientific purposes). Procedures were approved by the Ethical Committee of Animal Experimentation of the CIEMAT in accordance with all external and internal bio-safety and bio-ethics guidelines.

### Generation and characterization of mouse Ad-MSCs

Adipose tissue-derived mesenchymal stem cells (Ad-MSCs) were generated from B6D2F1 mice epiploon adipose tissue. Fat was cut into pieces, digested for 2 h with 1 mg/ml collagenase A (Roche Diagnostics GmbH, Mannheim, Germany) in DMEM, centrifuged, and filtered through 40-μm filters. Samples were then cultured at 37 °C, 5 % CO_2_ in flasks (Nalge Nunc International, Rochester, NY, USA) at 1.6×10^5^ cells/cm^2^, in MesenCult medium plus supplements for mouse cells (Stemcell Technologies, Vancouver, BC, Canada). After 24 h, non-adherent cells were discarded. Fresh medium was added and replaced twice a week. At 80 % confluence, adherent cells were trypsinized, washed, and seeded at 4×10^3^ cells/cm^2^. In all the experiments, Ad-MSCs were used at 5–8 passages.

The osteogenic and adipogenic Ad-MSC differentiation capacity was demonstrated by using StemXVivo™ Osteogenic/Adipogenic Base Media Supplemented with StemXVivo™ Osteogenic Supplement or StemXVivo™ Adipogenic Supplement (R&D Systems, Minneapolis, MN, USA), respectively, in accordance with the instructions of the manufacturer.

For immunophenotype analysis, Ad-MSCs were harvested with 0.05 % trypsin-EDTA, washed, resuspended in 1 % bovine serum albumin (Sigma-Aldrich, St. Louis, MO, USA) supplemented phosphate-buffered saline (PBS), incubated 30 m at 4 °C with monoclonal antibodies, and washed. The flow cytometry analysis included CD34, CD45.1, CD45.2, CD90, CD117, CD105, CD73, CD166, and CD29 expression (BD Biosciences Pharmingen, San Diego, CA, USA). Results were analyzed with FlowJo Analysis (Tree Star, now part of FlowJo LLC, Ashland, OR, USA).

### Hematopoietic stem cell purification

BM cells were isolated from tibias and femurs of P3D2F1 mice by flushing DMEM into each bone. Cells were subjected to red blood cells lysis and resuspended in PBS. Purified HSCs, LSK (lineage-negative, Sca-1^+^, cKit^+^) cells, were obtained by whole BM cell sorting by using lineage-specific antibodies (anti-Gr1, anti-B220, anti-CD3e, anti-CD11b, anti-Ter119) and anti-Sca-1 and anti-cKit antibodies (BD Biosciences). LSK cells were sorted by using a BD Influx cell sorter (BD Biosciences). After cell sorting, 90.75 ± 5 % LSK cells purities were obtained.

### Hematopoietic stem cell transplants

Donor hematopoietic cells were obtained from P3D2F1 mice (CD45.1/CD45.2), while B6D2F1 (CD45.2) mice were always used as recipients. In some experiments, BM cells from primary recipients were re-transplanted into secondary and tertiary recipients. Twenty-four hours before hematopoietic transplantation was performed, recipient mice were irradiated with x-ray equipment MG324 (300 kV, 12.8 mA; Philips, Hamburg, Germany).

Donor cell engraftment was analyzed by flow cytometry at different times post-transplantation by analyzing the proportion of CD45.1^+^ and CD45.2^+^ cells. Peripheral blood (PB) or BM cells were stained with anti-CD45.1 and anti-CD45.2 (BioLegend, San Diego, CA, USA) mouse monoclonal antibodies and analyzed after treating the cells with red blood cell lysis solution. Results were analyzed with FlowJo Analysis (Tree Star). All HSCT experiments were repeated at least three times. Each study group totaled 10–15 mice.

### Bio-distribution assays

LSK cells were stained with DiD (Invitrogen, Waltham, MA, USA) in accordance with the protocol of the manufacturer. Purified 10^4^ LSK cells, with or without 6×10^5^ Ad-MSCs, were intravenously infused in 5 Gy-irradiated B6D2F1 recipient mice. Mice were sacrificed 2, 4, and 24 h after transplant. Lungs, spleen, blood, and BM were analyzed by flow cytometry to determine the number of DiD^+^ cells in each organ. For BM, analyses were performed in cell suspensions generated by tibial and femoral BM. Total BM was estimated assuming that femoral and tibial BM correspond to 25 % of total BM [25].

### Duplets analysis

Ad-MSCs were detached from culture plates with 0.25 % Trypsine-EDTA (Gibco, Life Technologies, Carlsbad, CA, USA) and stained with carboxyfluorescein succinimidyl ester (CFSE) (Molecular Probes, Invitrogen). Similarly, purified LSK cells were stained with DiD (Molecular Probes, Invitrogen) in accordance with the protocol of the manufacturer. Ten minutes after the incubation of stained cells (6×10^5^ Ad-MSCs and 1500 LSK-DiD^+^CFSE^+^), single cell or duplet cell populations were analyzed by flow cytometry. This period was established considering the time in which Ad-MSCs and LSK cells were mixed prior to infusion in mice.

In some experiments, Ad-MSCs and LSK cells were analyzed by using the ImageStream100 flow cytometer (Amnis Corporation, Seattle, WA, USA) and images were collected on a six-channel charge-coupled device (CCD) digital camera. Each cell image was decomposed into six separate sub-images, each corresponding to a different color component.

### Statistical analysis

Statistical analysis was performed by one-way analysis of variance on rank with Tukey *post hoc* test. Results are expressed as mean ± standard error of the mean and were considered significant if the *P* value was not more than 0.05. Statistical analysis was performed with the GraphPad Prism 5.00 for Windows (GraphPad Software, Inc., La Jolla, CA, USA).

## Results

### Ad-MSCs enhance the engraftment of purified HSCs in congenic recipients conditioned with non-myeloablative radiation

**Fig. 1:**
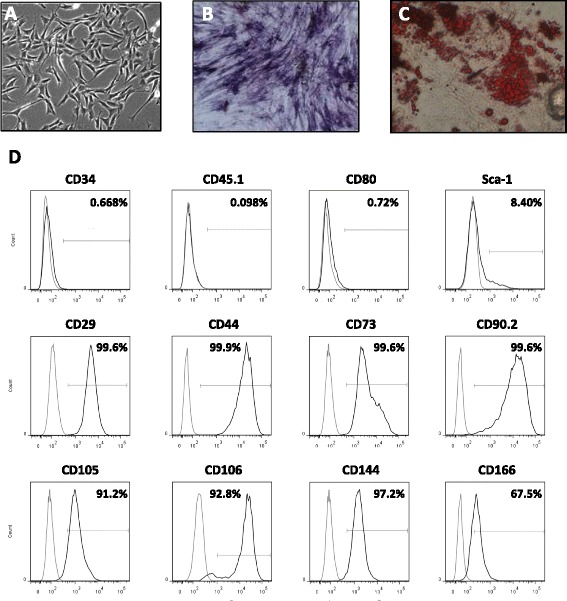
Mouse Ad-MSC characterization. **a** Ad-MSCs’ fibroblast-like morphology. **b** Osteogenic differentiation. **c** Adipogenic differentiation. **d** Ad-MSC immunophenotype, negative for hematopoietic markers CD34, CD45.1, CD80, and low Sca-1 and positive for CD29, CD44, CD73, CD90.2, CD105, CD106, CD144, and CD166 expression. *Ad-MSC* adipose tissue-derived mesenchymal stem cell

In the current studies, MSCs obtained from adipose tissue (Ad-MSCs) were used throughout. Consistent with our previous studies, Ad-MSCs showed the characteristic fibroblast-like morphology (Fig. 1a), had osteogenic and adipogenic differentiation capacity (Fig. 1b, c), and were negative for hematopoietic marker expression and positive for CD29, CD44, CD73, CD90.2, CD105, CD106, CD144, and CD166 expression (Fig. 1d).

**Fig. 2:**
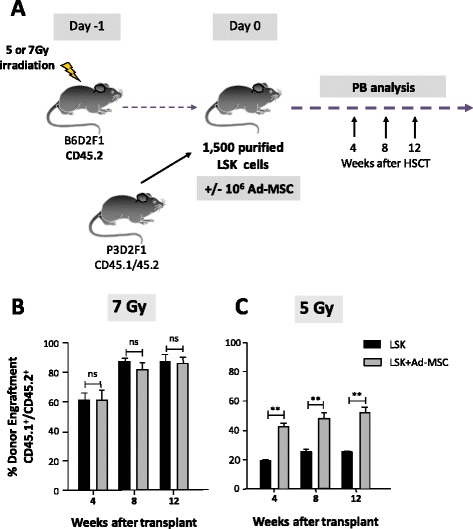
Influence of Ad-MSC co-infusion on HSC engraftment in congenic recipients conditioned with non-myeloablative irradiation. **a** CD45.2^+^ recipient mice were transplanted with 1500 purified donor CD45.1^+^/CD45.2^+^ LSK cells, with or without 10^6^ Ad-MSCs co-infusion. Donor cell engraftment in peripheral blood of recipients conditioned with **b** 7 Gy or **c** 5 Gy. Bars represent standard error of the mean. **P ≤ 0.01. *Ad-MSC* adipose tissue-derived mesenchymal stem cell, *HSC* hematopoietic stem cell, *HSCT* hematopoietic stem cell transplant, *LSK* lineage^−^ Sca-1^+^ cKit^+^, *ns* non-significant difference

To investigate the impact of Ad-MSCs on HSC engraftment, sub-lethally irradiated recipient mice (CD45.2/CD45.2) were transplanted with 1500 purified LSK cells from congenic (CD45.1/CD45.2) mice, either with or without Ad-MSCs (10^6^ Ad-MSCs per mouse) (Fig. 2a).

In a first set of experiments, recipient mice were irradiated with a relatively high dose of 7 Gy. As shown in Fig. 2b, high levels of donor hematopoietic engraftment were observed in all instances, and no differences of engraftment could be found between mice infused with or without Ad-MSCs.

In a second set of experiments, recipient mice were conditioned with a lower radiation dose of 5 Gy. Under these radiation conditions, the co-infusion of Ad-MSCs with the LSK markedly increased the engraftment of the LSK cells (Fig. 2c). As shown in the figure, differences remained significant and stable throughout the 12-week post-transplantation period of analysis. These results show that Ad-MSCs improve the engraftment of purified LSK cells, particularly in recipients subjected to mild conditioning.

### The hematopoietic engraftment-supportive effect of Ad-MSCs is dose-dependent

**Fig. 3:**
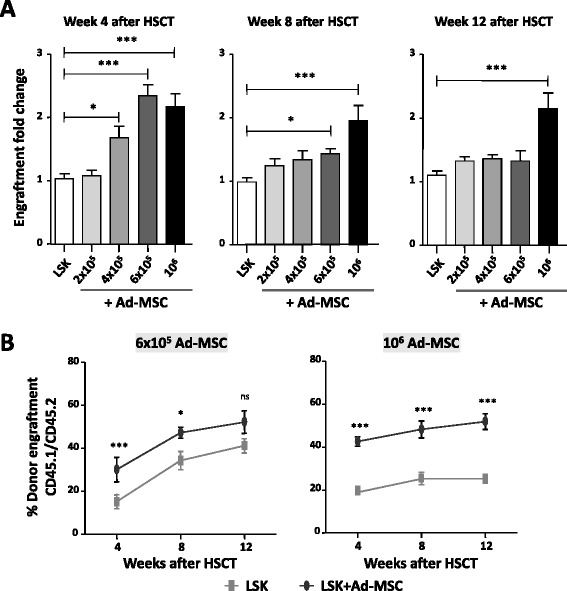
Ad-MSC dose-dependent effect on HSC engraftment transplanted into congenic recipients conditioned with moderate radiation. **a** Fold-increase of donor hematopoietic engraftment in peripheral blood of 5 Gy-irradiated recipient mice transplanted with 1500 LSK cells co-infused with increasing numbers of Ad-MSCs. **b** Evolution of peripheral blood donor engraftment in the peripheral blood of recipient mice co-infused with 1500 LSK plus 6×10^5^ or 10^6^ Ad-MSCs. Data obtained at 4, 8, and 12 weeks post-transplantation are shown. Bars represent standard error of the mean. *P ≤ 0.05. ***P ≤ 0.001. *Ad-MSC* adipose tissue-derived mesenchymal stem cell, *HSC* hematopoietic stem cell, *HSCT* hematopoietic stem cell transplant, *LSK* lineage^−^ Sca-1^+^ cKit^+^, *ns* non-significant difference

To investigate whether the engraftment effect of Ad-MSCs was dose-dependent, 1500 LSK cells were co-infused with increasing numbers of Ad-MSCs (from 2×10^5^ Ad-MSCs to 10^6^ Ad-MSCs/mouse) in 5 Gy-irradiated recipients (Fig. 3). Analyses performed 4 weeks after transplant showed that the co-infusion of 2×10^5^ Ad-MSCs did not increase the engraftment of LSK cells, as compared with data obtained when the LSK cells were transplanted as a sole population. In contrast to this data, the co-infusion of 4×10^5^ Ad-MSCs mediated a significant increase in the engraftment of LSK cells, an observation that was even more marked when 6×10^5^ Ad-MSCs/mouse were co-infused. No further increments were observed in the group co-infused with 10^6^ Ad-MSCs (Fig. 3a). When analyses were performed at 8 and 12 weeks after transplant, doses of 6×10^5^ and 10^6^ Ad-MSCs still showed improvements in the engraftment of purified LSK cells (see fold increases of engraftment in Fig. 3a and representative absolute values of engraftment in Fig. 3b). These results thus demonstrate that the hematopoietic engraftment effect of Ad-MSCs is dose-dependent. Additionally, our data indicate that relatively low doses of Ad-MSCs can accelerate the engraftment of transplanted LSK cells but that higher Ad-MSC doses are required to improve LSK cell engraftment in later stages post-transplantation.

### Ad-MSCs improve the engraftment of long-term hematopoietic repopulating cells

In subsequent experiments, we investigated whether the engraftment effect of Ad-MSCs was applicable not only to short-term repopulating progenitor cells but also to true HSCs. As in our previous experiments, in these studies 5 Gy-irradiated recipients were transplanted with 1500 LSK cells, either with or without 6×10^5^ Ad-MSCs. Six months after transplantation, BM cells from each of the two recipient groups were pooled and then re-transplanted into secondary recipients that had been conditioned with 11 Gy, to prevent endogenous reconstitution. Three months later, BM cells from secondary recipients were further transplanted into tertiary recipients also conditioned with 11 Gy.

**Fig. 4:**
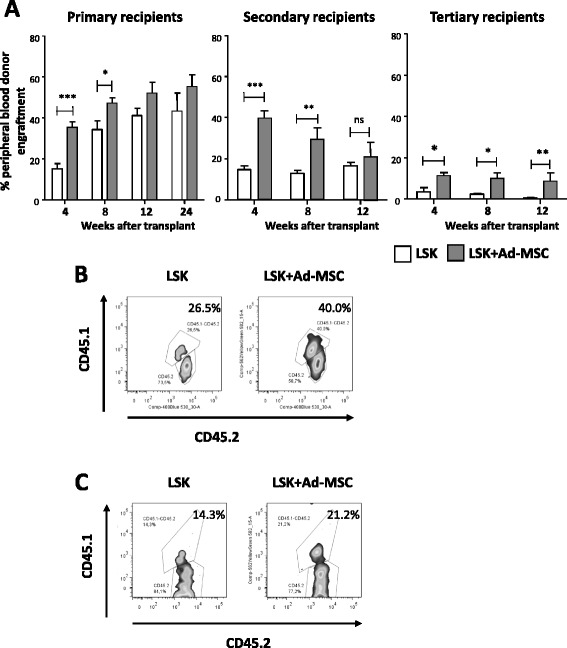
Short-term and long-term hematopoietic engraftment of recipient mice co-infused with purified HSCs and Ad-MSCs. **a** Donor CD45.1^+^/CD45.2^+^ cell engraftment detected in CD45.2^+^ recipients’ peripheral blood. Five gray-irradiated primary recipients were infused 1500 LSK, with (*gray bars*) or without (*white bars*) 6×10^5^ Ad-MSC co-infusion. Secondary and tertiary recipients were irradiated with 11 Gy and transplanted with pooled 2×10^6^ BM cells obtained 24 weeks after HSCT from the primary recipients and at 12 weeks after HSCT from the secondary recipients. **b** Flow cytometry analysis showing the percentage of LSK cells that were of donor CD45.1^+^/CD45.2^+^ origin in pooled BM samples from primary recipients (CD45.2^+^) 24 weeks after transplantation. **c** Similar analyses conducted in pooled BM samples from secondary recipients (CD45.2^+^) 12 weeks after transplantation. Bars represent standard error of the mean. *P ≤ 0.05. **P ≤ 0.01. ***P ≤ 0.001. *Ad-MSC* adipose tissue-derived mesenchymal stem cell, *BM* bone marrow, *HSC* hematopoietic stem cell, *HSCT* hematopoietic stem cell transplant, *LSK* lineage^−^ Sca-1^+^ cKit^+^, *ns* non-significant difference

As observed in previous experiments (Fig. 3), a dose of 6×10^5^ Ad-MSCs significantly increased LSK engraftment in primary recipients during the first weeks after transplant, although at 12 and 24 weeks post-transplantation differences did not reach statistical significance (Fig. 4a). This figure also shows that most of the PB analyses performed in secondary and tertiary recipients revealed improved donor engraftments in the LSK + Ad-MSC group, as compared with the LSK group. Consistent with these observations, the proportion of precursor cells that were of donor origin (percentage of LSK cells that were CD45.1^+^/CD45.2^+^) in pooled BM samples that were transplanted in secondary and tertiary recipients was also higher in the LSK + Ad-MSC groups (Fig. 4b, c). These experiments allow us to conclude that the co-infusion of Ad-MSCs improves the engraftment not only of progenitors responsible for the early stages of hematopoietic engraftment but also of long-term repopulating HSCs.

### Ad-MSCs improve the homing of transplanted LSK cells into the BM of recipient mice

To elucidate whether the increased engraftment of LSK cells associated with Ad-MSC co-infusion was due to an improved homing of transplanted LSK cells into recipient BM, 10^4^ LSK cells stained with a vital dye (DiD) were transplanted, either with or without 6×10^5^ Ad-MSCs/mouse, into 5 Gy-irradiated recipients. At 2, 4, and 24 h after infusion, the percentage of the transplanted DiD^+^ LSK cells present in femoral BM, and by extrapolation in the whole BM, as well as in PB, spleen, and lung, was determined.

**Fig. 5:**
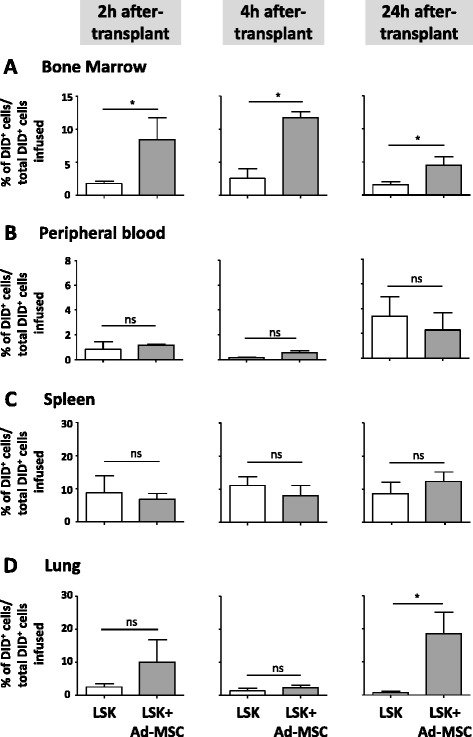
Effect of Ad-MSC co-infusion in the homing of transplanted HSCs in the recipients’ organs. Two, four, and 24 hours after the infusion of DiD^+^ LSK cells, the number present in **a** bone marrow, **b** peripheral blood, **c** spleen, and **d** lungs of recipient mice was determined. The figure represents the proportion of the infused DiD^+^ LSK cells detected in each organ. *White bars*, mice infused with LSK cells. *Grey bars*, mice co-infused with LSK cells and Ad-MSCs. Bars represent standard error of the mean. *P ≤ 0.05. *Ad-MSC* adipose tissue-derived mesenchymal stem cell, *HSC* hematopoietic stem cell, *LSK* lineage^−^ Sca-1^+^ cKit^+^, *ns* non-significant difference

Interestingly, Ad-MSC co-infusion significantly increased the homing of LSK cells in recipients’ BM at all investigated time points (2, 4, and 24 h) (Fig. 5a). Neither in PB (Fig. 5b) nor in the spleen (Fig. 5c) did Ad-MSC co-infusion modify the presence of LSK cells in these tissues. However, at 24 h post-transplantation, increased numbers of donor LSK cells were observed in the lung of recipients that had been transplanted with Ad-MSCs (Fig. 5d), indicating that the increased homing of LSK cells in BM was not due to a decreased trapping of these cells in the lung.

These results strongly suggest that the improved engraftment of LSK cells associated with Ad-MSC co-infusion, and thus the accelerated and sustained hematopoietic reconstitution of transplanted recipients, is mediated by the improved homing of transplanted precursor cells and HSCs in recipient BM.

### Ad-MSC-HSC co-infusion is essential for the supportive hematopoietic engraftment effect of Ad-MSCs

**Fig. 6:**
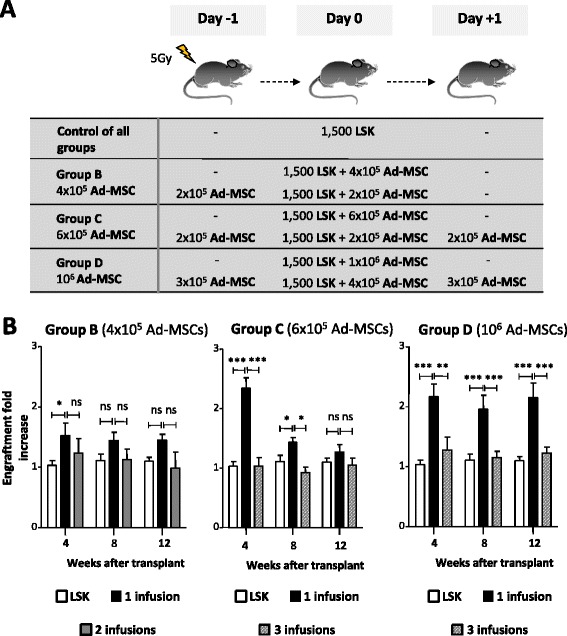
Influence of Ad-MSC dose fractionation on the engraftment of purified HSCs. **a** Illustration of the experimental protocol corresponding to results shown in **b**. As a control group, 5 Gy-irradiated mice were intravenously infused with 1500 LSK cells on day 0. Three study groups received one to three different Ad-MSC doses intravenously co-infused, following different administration schemes, depicted in the figure’s experimental design. **b** Analysis of the donor cell engraftment (fold-increase) with respect to mice transplanted with 1500 LSK cells. Bars represent standard error of the mean. *P ≤ 0.05. ***P ≤ 0.001. *Ad-MSC* adipose tissue-derived mesenchymal stem cell, *HSC* hematopoietic stem cell, *LSK* lineage^−^ Sca-1^+^ cKit^+^, *ns* non-significant difference

To investigate whether the HSC engraftment effects mediated by Ad-MSCs require their co-infusion with the LSK cells, two different sets of experiments were conducted. In the first experiments (Fig. 6), a group of recipient mice was co-infused with 1500 purified LSK cells together with Ad-MSCs, as in our previous experiments. Additional groups of transplanted mice were now included, in which the total Ad-MSC dose was split into up to three infusions that were administered either the day before or both the day before and the day after the co-infusion of LSK and Ad-MSCs (see experimental protocol in Fig. 6a). As shown in Fig. 6b, doses of 6×10^5^ and 10^6^ Ad-MSCs—which markedly improved the engraftment of 1500 LSK cells when co-infused in a single dose—had no effect when divided into three administrations, and only one of them co-infused with the LSK cells. These studies thus indicate that the total effective dose of Ad-MSCs need to be infused with the LSK cells at the moment of the LSK infusion but not earlier or later than LSK infusion.

**Fig. 7:**
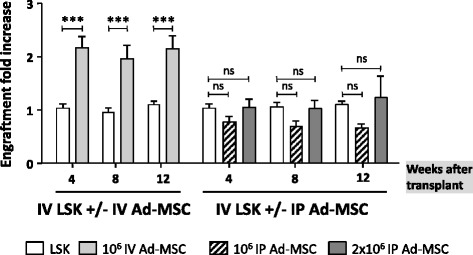
Influence of Ad-MSC administration route on the engraftment of purified HSCs. Donor cell engraftment (fold increase) in the peripheral blood of recipient mice intravenously infused with 1500 LSK cells (*white bars*), with intravenous co-infusion of 10^6^ Ad-MSCs (*gray bars*), or with intraperitoneal injection of 10^6^ (*striped bars*) or 2×10^6^ (*dark gray bars*) Ad-MSCs. Bars represent standard error of the mean. ***P ≤ 0.001. *Ad-MSC* adipose tissue-derived mesenchymal stem cell, *HSC* hematopoietic stem cell, *IP* intraperitoneal injection, *IV* intravenous infusion, *LSK* lineage^−^ Sca-1^+^ cKit^+^, *ns* non-significant difference

In subsequent experiments represented in Fig. 7, the beneficial engraftment effect of Ad-MSCs was investigated in recipients in which Ad-MSCs (10^6^ cells) were IV co-infused with the LSK cells, with respect to recipients in which the Ad-MSCs were infused via intraperitoneal (IP) injection simultaneously to the IV injection of LSK cells. In previous studies, we demonstrated that an anti-GVHD effect of IV-injected Ad-MSCs is reproduced by IP-administered Ad-MSCs if the cell dose was twofold increased [26]. In our current experiments, doses of 10^6^ or 2×10^6^ Ad-MSCs were, therefore, considered.

In contrast with data obtained in mice IV co-infused with LSK and Ad-MSCs, the IP injection of Ad-MSCs did not mediate any significant effect upon the engraftment of IV infused LSK cells (Fig. 7). These experiments strongly suggest that the supportive hematopoietic engraftment effect of Ad-MSCs is not paracrine but rather is due to the direct contact between the LSK cells and the Ad-MSCs.

**Fig. 8:**
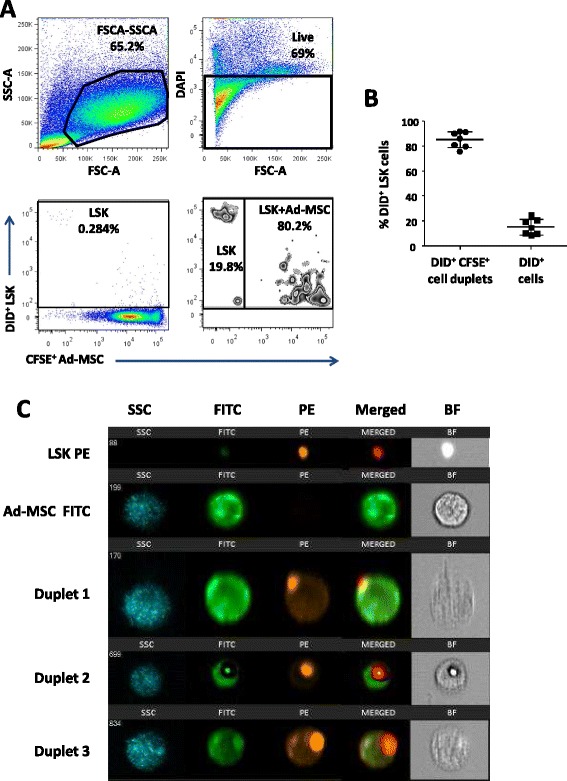
Flow cytometry analysis of the rapid *in vitro* interaction of HSCs with Ad-MSCs. **a** Representative flow cytometry duplet analysis of 6×10^5^ CFSE-stained Ad-MSCs and 1500 DiD-stained LSK cells, 10 minutes after co-culture. **b** Percentage of DiD^+^LSK cells forming duplets with CFSE^+^ Ad-MSCs detected in the different assays. **c** Multi-spectral images of PE^+^ LSK cells and FITC^+^ Ad-MSCs. Individual cells and three representative duplets are shown by a row of images that include side scatter signal (*blue*), FITC signal (*green*), PE signal (*orange*), and FITC and PE signals together (*merged column*). Bright-field (BF) of each cell sample is also represented. *Ad-MSC* adipose tissue-derived mesenchymal stem cell, *CFSE* carboxyfluorescein succinimidyl ester, *DAPI* 4',6-diamidino-2-phenylindole, *FITC* fluorescein isothiocyanate, *FSC* forward scatter, *HSC* hematopoietic stem cell, *LSK* lineage^−^ Sca-1^+^ cKit^+^, *PE* phycoerythrin, *SSC* side scatter

This hypothesis was further supported by the finding that after a short incubation (10 minutes) of DiD^+^ LSK cells with CSFE^+^ Ad-MSCs, 83.69 ± 2 % of the LSK cells formed DiD^+^/CFSE^+^ cell duplets, showing the close interaction between both cell types (Fig. 8a, b). In similar experiments, co-incubated PE^+^ LSK and fluorescein isothiocyanate-positive (FITC^+^) Ad-MSCs were analyzed by image stream flow cytometry methods to examine the direct interaction of the two cell populations. In Fig. 8c, the intimate association of LSK and Ad-MSCs is shown. These final observations suggest that Ad-MSCs may act as LSK carriers *in vivo*, thus facilitating the migration and homing of the HSC and hematopoietic progenitor cells into the BM niche during the early stages after transplantation.

## Discussion

Previous studies have shown that MSCs have important immunosuppressive properties [27–29] and are constitutive of the BM microenvironment, where they secrete factors involved in HSC maintenance [2, 3, 30]. Different studies have demonstrated a beneficial MSC effect on HSC engraftment in xenogeneic transplants in NOD/SCID mice [19, 20]. In humans, MSCs have been used to improve or accelerate (or both) the hematopoietic engraftment in different allogeneic HSCT settings with risk of engraftment failure [21, 23, 31]. In these case reports, the proposed hematopoietic engraftment effects of MSCs are commonly associated with the immunosuppressive effects of MSCs, which may limit the immune reactions between host and donor cells. However, the question of whether other, non-immunological effects could play a role in the hematopoietic engraftment effect mediated by MSCs has never been investigated, even in the context of autologous transplantation.

We have, therefore, investigated the hematopoietic engraftment effect of Ad-MSCs by using an autologous hematopoietic transplantation model based on the infusion of donor CD45.1^+^/CD45.2^+^ LSK cells into congenic CD45.2 recipient mice. In this study, Ad-MSCs were used throughout since these cells are more easily obtained in large quantities than BM-MSCs and share similar phenotypic and functional characteristics [28, 32, 33].

Our first experiments showed that the co-infusion of Ad-MSCs did not have any impact on the engraftment of purified LSK cells if these cells are transplanted into recipients conditioned with a relatively high dose of 7 Gy, a radiation dose associated with high levels of engraftment because of the very high mortality of the recipient’s endogenous hematopoiesis. In contrast with this observation, when similar experiments were conducted in recipients conditioned with a lower dose of 5 Gy, a significant increase in the engraftment of purified LSK cells was induced by Ad-MSCs.

Consistent with previous observations in NOD/SCID mice transplanted with human CD34^+^ cells [20], an evident Ad-MSC dose-dependent hematopoietic engraftment effect was observed in our autologous transplantation model. This increase was more significant during the first few weeks after HSCT, a period which is particularly critical after transplantation. In subsequent experiments based on the serial BM transplantations, we demonstrate that Ad-MSCs improve the engraftment not only of the short-term repopulating cells but also of the true HSCs.

One of the possible mechanisms underlying the supporting engraftment effect of Ad-MSCs could be related to the numerous cytokines and growth factors secreted by these cells, which may increase the proliferation of engrafted hematopoietic precursors. Alternatively, Ad-MSCs could improve the homing of transplanted HSCs in the BM niches. This hypothesis was confirmed by our experiments represented in Fig. 5, which demonstrate that Ad-MSCs increase the homing of transplanted LSK cells in recipients’ BM during the early stages post-infusion.

To elucidate whether paracrine-mediated Ad-MSC effects could account for the improved engraftments observed in transplanted recipients, Ad-MSCs administration was either fractioned in up to three different consecutive infusions or injected via IP, instead of intravenously co-infused with the LSK cells. None of these Ad-MSC administration approaches facilitated the engraftment of purified LSK cells. This contrasts our earlier studies [26], in which the IP administration of Ad-MSCs was able to control GVHD in mice transplanted with allogeneic cells, indicating that soluble factors released by the Ad-MSCs are key immune-suppressors of the GVHD. In our current studies, the IV co-infusion of Ad-MSC/LSK was mandatory to mediate a significant hematopoietic engraftment effect. These results, together with the observation that after only 10 min of LSK and Ad-MSC incubation most of LSK cells co-localize with Ad-MSCs, strongly suggest that the LSK/Ad-MSCs interaction is a requisite to improve the engraftment of transplanted HSCs.

Whether or not other sources of MSCs share the hematopoietic engraftment ability now described for Ad-MSCs is currently unknown. However, observations showing similar phenotypic and functional properties in MSCs obtained from different sources [32] would suggest that the effects reported in our study may not be specific for Ad-MSCs.

The results obtained in this study would have a practical application in the field of HSC gene therapy, particularly for the treatment of diseases, such as Fanconi anemia, in which the collection of clinically relevant numbers of HSCs constitutes one of the bottlenecks to restore patient hematopoiesis with autologous gene-corrected HSCs [34, 35]. Improving the engraftment of autologous HSCs by MSC co-infusion would have further advantages in several other gene therapy applications in which corrected HSCs do not develop a proliferation advantage over uncorrected HSCs [36–39]. Moreover, the observation that the HSC engraftment effect of MSCs is most significant when moderate conditioning is used further suggests that MSC co-infusion would help to minimize toxic conditioning regimens currently used in many HSC gene therapy protocols [36–39].

## Conclusions

Taken together, our data demonstrate for the first time in an autologous mouse transplantation model that Ad-MSCs improve the homing of donor hematopoietic stem and progenitor cells into recipient BM niches, facilitating the stable reconstitution of transplanted recipients with infused hematopoietic grafts. The conclusions of this study would have a practical application in the field of HSC transplants, including gene therapy.

## Abbreviations

Ad-MSC: Adipose tissue-derived mesenchymal stem cell
BM: Bone marrow
CFSE: Carboxyfluorescein succinimidyl ester
CIEMAT: Centro de Investigaciones Energéticas, Medioambientales y Tecnológicas
DMEM: Dulbecco’s modified Eagle’s medium
GVHD: Graft-versus-host disease
HSC: Hematopoietic stem cell
HSCT: Hematopoietic stem cell transplant
IP: Intraperitoneal
IV: Intravenous
LSK: Lineage^−^ Sca-1^+^ cKit^+^
MSC: Mesenchymal stem cell
NOD/SCID: Non-obese diabetic/severe combined immunodeficiency
PB: Peripheral blood
PBS: Phosphate-buffered saline
PE: Phycoerythrin

## References

[1] Friedenstein AJ, Latzinik NW, Grosheva AG, Gorskaya UF (1982). Marrow microenvironment transfer by heterotopic transplantation of freshly isolated and cultured cells in porous sponges. Exp Hematol..

[2] Pittenger MF, Mackay AM, Beck SC, Jaiswal RK, Douglas R, Mosca JD (1999). Multilineage potential of adult human mesenchymal stem cells. Science..

[3] Conget PA, Minguell JJ (1999). Phenotypical and functional properties of human bone marrow mesenchymal progenitor cells. J Cell Physiol..

[4] Le Blanc K (2003). Immunomodulatory effects of fetal and adult mesenchymal stem cells. Cytotherapy..

[5] Yanez R, Oviedo A, Aldea M, Bueren JA, Lamana ML (2010). Prostaglandin E2 plays a key role in the immunosuppressive properties of adipose and bone marrow tissue-derived mesenchymal stromal cells. Exp Cell Res..

[6] Aggarwal S, Pittenger MF (2005). Human mesenchymal stem cells modulate allogeneic immune cell responses. Blood..

[7] Bernardo ME, Fibbe WE (2013). Mesenchymal stromal cells: sensors and switchers of inflammation. Cell Stem Cell..

[8] Le Blanc K, Frassoni F, Ball L, Locatelli F, Roelofs H, Lewis I (2008). Mesenchymal stem cells for treatment of steroid-resistant, severe, acute graft-versus-host disease: a phase II study. Lancet..

[9] Ball LM, Bernardo ME, Roelofs H, van Tol MJ, Contoli B, Zwaginga JJ (2013). Multiple infusions of mesenchymal stromal cells induce sustained remission in children with steroid-refractory, grade III-IV acute graft-versus-host disease. Br J Haematol..

[10] Karussis D, Karageorgiou C, Vaknin-Dembinsky A, Gowda-Kurkalli B, Gomori JM, Kassis I (2010). Safety and immunological effects of mesenchymal stem cell transplantation in patients with multiple sclerosis and amyotrophic lateral sclerosis. Arch Neurol..

[11] Connick P, Kolappan M, Crawley C, Webber DJ, Patani R, Michell AW (2012). Autologous mesenchymal stem cells for the treatment of secondary progressive multiple sclerosis: an open-label phase 2a proof-of-concept study. Lancet Neurol..

[12] Garcia-Olmo D, Garcia-Arranz M, Herreros D, Pascual I, Peiro C, Rodriguez-Montes JA (2005). A phase I clinical trial of the treatment of Crohn’s fistula by adipose mesenchymal stem cell transplantation. Dis Colon Rectum..

[13] Lee WY, Park KJ, Cho YB, Yoon SN, Song KH, Kim do S (2013). Autologous adipose tissue-derived stem cells treatment demonstrated favorable and sustainable therapeutic effect for Crohn’s fistula. Stem Cells.

[14] Horwitz EM, Gordon PL, Koo WK, Marx JC, Neel MD, McNall RY (2002). Isolated allogeneic bone marrow-derived mesenchymal cells engraft and stimulate growth in children with osteogenesis imperfecta: Implications for cell therapy of bone. Proc Natl Acad Sci U S A..

[15] Orozco L, Munar A, Soler R, Alberca M, Soler F, Huguet M (2013). Treatment of knee osteoarthritis with autologous mesenchymal stem cells: a pilot study. Transplantation..

[16] Wakitani S, Mitsuoka T, Nakamura N, Toritsuka Y, Nakamura Y, Horibe S (2004). Autologous bone marrow stromal cell transplantation for repair of full-thickness articular cartilage defects in human patellae: two case reports. Cell transplantation..

[17] Hare JM, Traverse JH, Henry TD, Dib N, Strumpf RK, Schulman SP (2009). A randomized, double-blind, placebo-controlled, dose-escalation study of intravenous adult human mesenchymal stem cells (prochymal) after acute myocardial infarction. J Am Coll Cardiol..

[18] Prockop DJ, Brenner M, Fibbe WE, Horwitz E, Le Blanc K, Phinney DG (2010). Defining the risks of mesenchymal stromal cell therapy. Cytotherapy..

[19] Angelopoulou M, Novelli E, Grove JE, Rinder HM, Civin C, Cheng L (2003). Cotransplantation of human mesenchymal stem cells enhances human myelopoiesis and megakaryocytopoiesis in NOD/SCID mice. Exp Hematol..

[20] Park SK, Won JH, Kim HJ, Bae SB, Kim CK, Lee KT (2007). Co-transplantation of human mesenchymal stem cells promotes human CD34+ cells engraftment in a dose-dependent fashion in NOD/SCID mice. J Korean Med Sci..

[21] Le Blanc K, Samuelsson H, Gustafsson B, Remberger M, Sundberg B, Arvidson J (2007). Transplantation of mesenchymal stem cells to enhance engraftment of hematopoietic stem cells. Leukemia..

[22] Ball LM, Bernardo ME, Roelofs H, Lankester A, Cometa A, Egeler RM (2007). Cotransplantation of ex vivo expanded mesenchymal stem cells accelerates lymphocyte recovery and may reduce the risk of graft failure in haploidentical hematopoietic stem-cell transplantation. Blood..

[23] Fang B, Song Y, Li N, Li J, Zhao RC (2008). Cotransplantation of haploidentical mesenchymal stem cells to reduce the risk of graft failure in a patient with refractory severe aplastic anemia. Acta Haematol..

[24] Koc ON, Gerson SL, Cooper BW, Dyhouse SM, Haynesworth SE, Caplan AI (2000). Rapid hematopoietic recovery after coinfusion of autologous-blood stem cells and culture-expanded marrow mesenchymal stem cells in advanced breast cancer patients receiving high-dose chemotherapy. J Clin Oncol..

[25] Boggs DR (1984). The total marrow mass of the mouse: a simplified method of measurement. Am J Hematol..

[26] Oviedo A, Yanez R, Colmenero I, Aldea M, Rubio A, Bueren JA (2013). Reduced efficacy of mesenchymal stromal cells in preventing graft-versus-host disease in an in vivo model of haploidentical bone marrow transplant with leukemia. Cell Transplant..

[27] Le Blanc K, Ringden O (2005). Immunobiology of human mesenchymal stem cells and future use in hematopoietic stem cell transplantation. Biol Blood Marrow Transplant..

[28] Yanez R, Lamana ML, Garcia-Castro J, Colmenero I, Ramirez M, Bueren JA (2006). Adipose tissue-derived mesenchymal stem cells have in vivo immunosuppressive properties applicable for the control of the graft-versus-host disease. Stem Cells..

[29] Tolar J, Le Blanc K, Keating A, Blazar BR (2010). Concise review: hitting the right spot with mesenchymal stromal cells. Stem Cells..

[30] Mendez-Ferrer S, Battista M, Frenette PS (2010). Cooperation of beta- and beta-adrenergic receptors in hematopoietic progenitor cell mobilization. Ann N Y Acad Sci..

[31] Fang B, Song Y, Li N, Li J, Han Q, Zhao RC (2009). Mesenchymal stem cells for the treatment of refractory pure red cell aplasia after major ABO-incompatible hematopoietic stem cell transplantation. Ann Hematol..

[32] Garcia-Gomez I, Elvira G, Zapata AG, Lamana ML, Ramirez M, Castro JG (2010). Mesenchymal stem cells: biological properties and clinical applications. Expert Opin Biol Ther..

[33] Puissant B, Barreau C, Bourin P, Clavel C, Corre J, Bousquet C (2005). Immunomodulatory effect of human adipose tissue-derived adult stem cells: comparison with bone marrow mesenchymal stem cells. Br J Haematol..

[34] Croop JM, Cooper R, Fernandez C, Graves V, Kreissman S, Hanenberg H (2001). Mobilization and collection of peripheral blood CD34+ cells from patients with Fanconi anemia. Blood..

[35] Kelly PF, Radtke S, Kalle C, Balcik B, Bohn K, Mueller R (2007). Stem cell collection and gene transfer in fanconi anemia. Mol Ther..

[36] Ott MG, Schmidt M, Schwarzwaelder K, Stein S, Siler U, Koehl U (2006). Correction of X-linked chronic granulomatous disease by gene therapy, augmented by insertional activation of MDS1-EVI1, PRDM16 or SETBP1. Nat Med..

[37] Cavazzana-Calvo M, Payen E, Negre O, Wang G, Hehir K, Fusil F (2010). Transfusion independence and HMGA2 activation after gene therapy of human beta-thalassaemia. Nature..

[38] Biffi A, Montini E, Lorioli L, Cesani M, Fumagalli F, Plati T (2013). Lentiviral Hematopoietic Stem Cell Gene Therapy Benefits Metachromatic Leukodystrophy. Science..

[39] Aiuti A, Biasco L, Scaramuzza S, Ferrua F, Cicalese MP, Baricordi C (2013). Lentiviral Hematopoietic Stem Cell Gene Therapy in Patients with Wiskott-Aldrich Syndrome. Science..

